# An exploratory single arm study to evaluate the role of an Ayurvedic treatment protocol as a prerequisite for *in vitro* fertilization in women with diminished ovarian reserve incorporating multi-omics approaches: study protocol

**DOI:** 10.3389/fmed.2025.1666888

**Published:** 2025-11-25

**Authors:** Anjaly Muraleedharan, Avani Pillai, Bipin G. Nair, Hemavathi Shivapura Krishnarajabhatt, Chithra Ramachandran, Anjana Aji, Muralidharan Vanuopadath

**Affiliations:** 1 Department of Stri Roga and Prasuti Tantra (Gynecology and Obstetrics), Amrita School of Ayurveda, Amrita Vishwa Vidyapeetham, Kollam, Kerala, India; 2 Department of Reproductive Medicine and Surgery, Amrita Institute of Medical Sciences, Kochi, Kerala, India; 3 School of Biotechnology, Amrita Vishwa Vidyapeetham, Kollam, Kerala, India; 4 Department of Obstetrics and Gynaecology, Amrita Institute of Medical Sciences, Kochi, Kerala, India

**Keywords:** diminished ovarian reserve, multi-omics, anti mullerian hormone (AMH), antral follicle count (AFC), Integrative medicine, traditional medicine, *in vitro* fertilization, ayurveda

## Abstract

**Introduction:**

Ovarian reserve depicts the quality and quantity of oocytes remaining in the ovaries and gives an idea about ovarian function at a given time. Because of aging, ovarian reserve diminish physiologically. However, many women face a non-physiologic reduction of ovarian reserve, irrespective of age. To date, standard-of-care treatment options are not available to treat diminished ovarian reserve (DOR). Studies shows that DOR patients mostly undergo *in vitro* fertilization (IVF) with donor cycles. Hence, identifying effective treatment modalities for DOR is an area of great clinical relevance. This trial will investigate the role of an Ayurvedic treatment protocol in DOR patients prior to IVF. Reports suggest that the factors influencing oocyte maturation can be determined through multi-omics analysis of the follicular fluid. However, the impact of Ayurvedic interventions in improving therapeutic outcomes has yet to be explored in detail. Hence this study also aims to explore whether therapeutic targets can be identified through multi-omics analysis of the follicular fluid collected from the participants after Ayurvedic treatment.

**Materials and methods:**

An open label single arm trial will be conducted to explore the role of an Ayurveda treatment protocol as a prerequisite for IVF in women with DOR. Forty women diagnosed with DOR satisfying eligibility criteria will be recruited to the study. Out of them, thirty participants will be undergoing Ayurveda treatment prior to their next IVF, and ten participants will be assigned to control group for follicular fluid analysis. Within subject change in serum anti-mullerian hormone and antral follicle count will be the primary outcomes evaluated. The multiomics analysis of follicular fluid will be done in 20 participants recruited to the study (treatment group 10 and control group 10).

**Discussion:**

This multidisciplinary exploratory clinical trial will be the first study to explore the role of an Ayurvedic treatment protocol in managing DOR. The multi-omics approaches will be helpful in identifying potential biomarkers associated with treatment response. The information gained through the study might be useful in planning a safe and feasible pre-conception care for DOR patients undergoing IVF.

**Clinical trial registration:**

ctri.nic.in, identifier CTRI/2023/11/059872.

## Introduction

Infertility is a disorder that impacts an individual’s psychological and social aspects (1). The treatment of infertility is mainly focused on its cause. With the advancement in medical sciences, assisted reproductive technique (ART) can now overcome major issues causing infertility (2). But the successful outcome of all these treatment modalities is completely based on one’s ovarian reserve. Ovarian reserve depicts the quality and quantity of oocytes remaining in the ovaries (3). It thus gives an idea about ovarian function or reproductive age at a given time. Because of aging, ovarian reserve diminish physiologically. But many women face a non-physiologic reduction of ovarian reserve, irrespective of age. The non-physiological reduction may result from multiple factors like autoimmune diseases, environmental pollution, psychological factors, genetic factors, endometriosis, pelvic inflammatory disease and chemotherapy (3). It has been reported that DOR is one of the leading reasons for seeking ART, with approximately 26% of patients undergoing ART due to this condition (4). DOR may be associated with irregular menstruation, hypomenorrhea, amenorrhea, short follicular phase, and poor ovarian response (5). Clinical assessments of DOR are done through ultrasonography by counting the number of ovarian antral follicles (AFC), and serum anti-mullerian hormone (AMH) test (6). DOR patients are being managed through conventional medicine by different stimulation protocols, Hormone replacement therapy (HRT), and Dehydroepiandrosterone (DHEA) as pre-treatment to IVF. It was observed that even after ovarian hyperstimulation with high-dose gonadotrophin, DOR patients have a smaller number of oocytes, a low fertilization rate, and a higher cycle cancellation rate (7). Most patients are ultimately left with the only option of IVF with a donor egg (8). But proper evidence is lacking to support their role in DOR (9). Also, the mode of action of these medicines is still unknown (10). Hence, identifying effective treatment modalities for DOR becomes an area of great clinical relevance.

Although there is no direct explanation of DOR in Ayurvedic texts, the clinical manifestations of certain gynaecological conditions show remarkable similarity. One such disorder is ārtavakṣaya, a menstrual abnormality arising from the depletion (kṣaya) of ārtava upadhātu (the supportive component of reproductive system), which closely parallels DOR in both its etiology and presentation. The clinical presentations of ārtavakṣaya is also evident in several endocrine disorders such as polycystic ovarian syndrome (PCOS), hyperprolactinemia, and thyroid dysfunction, as well as in physiological states like the premenopausal period, DOR, and premature ovarian failure (POF). Regardless of the underlying cause, these conditions share a common pathophysiological mechanism which involves dysregulation of the hypothalamo- pituitary -ovarian axis resulting in oligo or anovulation and manifesting clinically as oligomenorrhea or hypomenorrhea.

Ayurveda similarly attributes ārtavakṣaya to either qualitative or quantitative reduction of ārtava which possess agneya guna (fire element) characterized by yadocitakāla adarśana (irregular menstruation/oligomenorrhea), alpata (scanty menstruation/hypomenorrhea), and yonivedana (pain in vagina). The therapeutic approach in Ayurveda primarily aims to restore the function of ārtava. Management is two-fold, śodhana karma (cleansing therapy) and śamana karma (medicines for restoring normalcy or pacifying action). Śodhana may be achieved through vamana karma (therapeutic emesis) or virecana karma (therapeutic purgation), depending on the patient’s condition, followed by śamana therapy using medicines possessing agneya guṇa (to enhance fire element) (11). Ayurveda also emphasizes comprehensive preconception care which also includes basti karma (therapeutic enema) including uttarbasti (administration of therapeutic oil or ghee via vagina) after śodhana karma to optimize the quality of strībījam (ovum) and prepare the woman for healthy conception (12). In our clinical experience, several women diagnosed with DOR who underwent Ayurvedic interventions based on ārtavakṣaya cikitsā and preconception care protocols at our centre demonstrated notable improvement in serum AMH levels, subsequently becoming eligible for ART using their own oocytes (13). A few even achieved spontaneous conception during treatment. The group of formulations selected in this protocol was chosen for their established indications in restoring ārtava and facilitating preconception care. The specific medicines were selected based on each patient’s clinical condition and presentation. These encouraging outcomes underscore the potential role of Ayurveda in the management of DOR and highlight the need for generating the preliminary evidence for a safe and feasible Ayurveda treatment protocol. In the future, it will help to plan further clinical studies which may benefit DOR patients to have ART procedures with their eggs, thus opening a wide scope for research in integrated medicine.

In addition, the possibility of identifying detailed pharmacological mechanisms that might improve the therapeutic outcomes in DOR patients can be explored by incorporating multi-omics analysis. The role of proteomics, metabolomics, and transcriptomics in the analysis of human follicular fluid has been reported previously by several groups (14–16). Reports suggest that the factors influencing oocyte maturation can be determined by analysing the follicular fluid proteome (14, 17). Proteomics analysis of follicular fluid indicated the pharmacological mechanisms of Chinese patented drugs for improving DOR (18). Similarly, various groups have also identified serum metabolite markers for predicting poor ovarian reserve (19, 20) Also, transcriptomics analysis indicated that several genes are modulated in DOR patients (21). All these indicated that the pharmacological mechanisms induced by Ayurvedic treatment could be studied using multi-omics analysis to identify therapeutic targets. All these give an overall idea of how genes and proteins influence the quality of embryo and oocyte maturation, thereby improving therapeutic outcomes. However, the impact of Ayurvedic interventions in improving therapeutic outcomes has yet to be explored in detail. The pharmacological mechanisms induced by Ayurvedic treatment could be studied using multi-omics analysis to identify therapeutic targets, such as genes/proteins involved in oocyte development including steroidogenesis, oxidative stress, cumulus-oocyte signalling. Thus, the aim of this study is to explore the feasibility and within-subject changes in AMH/AFC and generate mechanistic hypotheses. The information gained through the study might be useful in planning future research which may help to incorporate a feasible Ayurvedic pre-conceptional care for DOR patients undergoing ART procedures. Similarly, the possibility of identifying therapeutic targets through multi-omics analysis can be studied.

## Materials and methods

**Figure 1:**
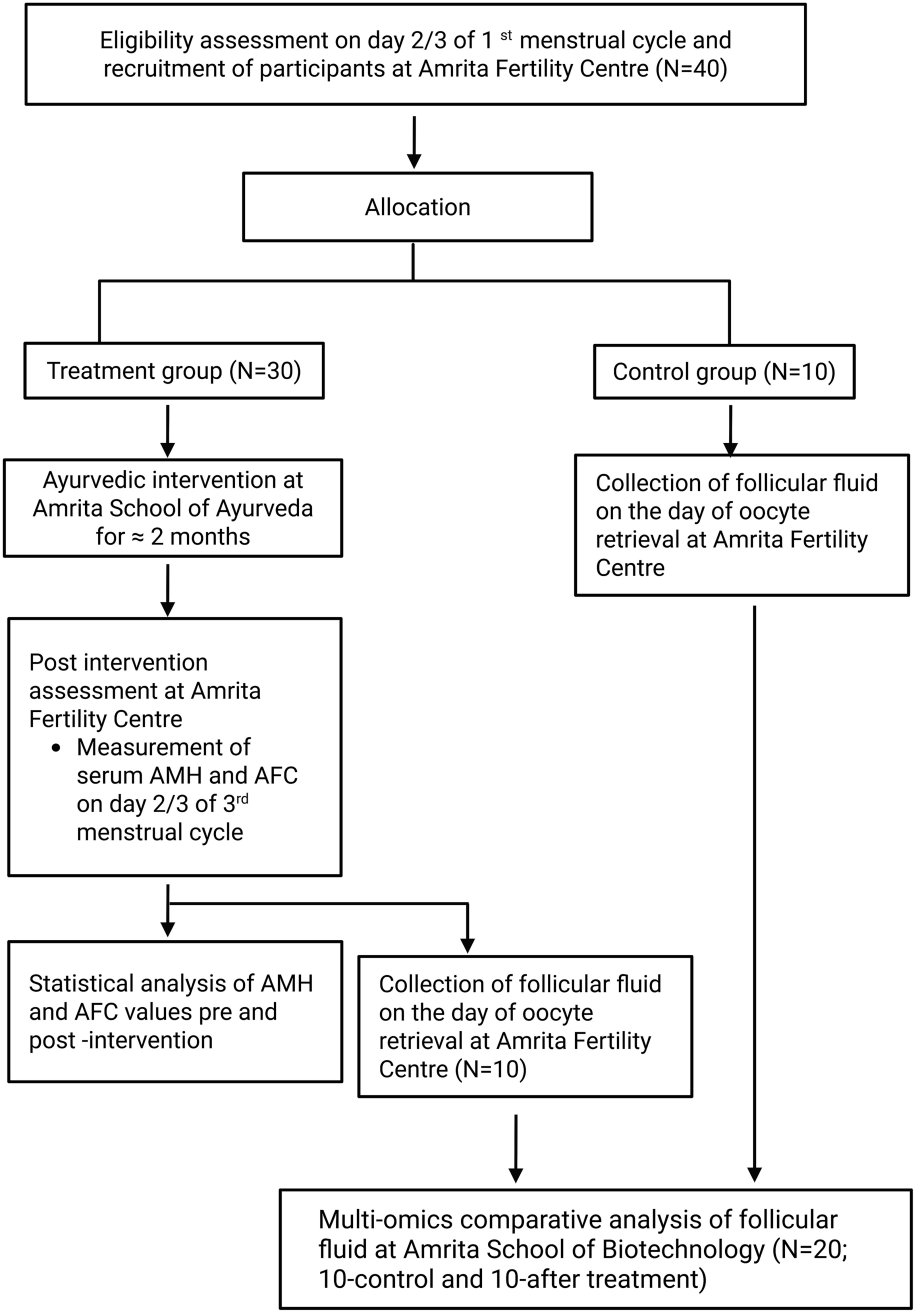
Trial participant’s flowchart.

This is a prospective, exploratory, single-arm, open-label, single-centred study, coordinated by Amrita Fertility Centre, Amrita School of Ayurveda, and Amrita School of Biotechnology, India with two assessment points (baseline and post-intervention). Participants are women diagnosed with DOR undergoing fertility treatments. Women under the age of 40 satisfying all the eligibility criteria and willing to provide informed consent will be recruited from Amrita Fertility Centre, Kochi. Recruited participants will be admitted at Amrita School of Ayurveda, Kollam to undergo a standardized Ayurvedic treatment protocol for a maximum period of 21 days. Following the inpatient treatment, participants will be advised to continue prescribed internal medications for an additional 1 month. After undergoing Ayurveda treatment, participants will be sent back to Amrita Fertility Centre for post-assessment and further fertility treatments, during which follicular fluid will be collected for multi-omics analysis. Figure 1 depicts the trial’s participant flow. The Standard Protocol Items: Recommendations for Intervention Trials (SPIRIT) declaration is followed while reporting the protocol (22). The institutional ethical committee approved the study and is prospectively registered under ctri.nic.in with the number CTRI/2023/11/059872 on November 15, 2023.

### Sample size

The sample size calculation was done using G*Power (version 3.1.9.7) (23) based on a previous study data. The initial sample size calculated to detect an effect size of 0.58 for a two-sided significance level of 0.05 and 80% power was 26. To account for feasibility considerations and an anticipated 20% dropout, the final sample size was set at 30 participants. Since the present study is planned as an exploratory study, this sample size is not meant to give confirmatory inference, rather this study is planned to provide preliminary data regarding the role of an Ayurvedic treatment protocol in women with reduced ovarian reserve.

Follicular fluid will be collected from 10 participants who undergo Ayurveda treatment and 10 control participants who is receiving a contemporaneous IVF stimulation protocol for performing multi-omics analysis (matched by age and baseline AMH/AFC). No formal sample size calculation was performed for the multi-omics analysis, as it is hypothesis-generating and intended for exploratory purposes. Because the 10 controls included are for exploratory multi-omics analysis only, no clinical inference will be drawn between treatment and control groups.

### Trial settings

The participants will be screened for eligibility at Amrita fertility centre, Kochi. After baseline assessment of AMH and AFC on day 2/3 of the menstrual cycle, 30 participants who are satisfying the eligibility criteria will be recruited for the study and will be admitted for Ayurveda treatment at Amrita School of Ayurveda, Kollam for a maximum of 21 days. On discharge, participants will be dispensed with internal medicines for 1 month. Post assessment will be done at Amrita Fertility Centre on day 2/3 of their next menstrual cycle which comes after completing 1 month of internal medications. Follicular fluid will be collected on the oocyte retrieval day of their next IVF cycle for multi-omics analysis (Treatment group—10 and Control group—10). The participant timeline is detailed in Table 1. The sample collected will be then transferred to Amrita School of Biotechnology for multi omics analysis.

**Table 1:** Participant timeline.

Time point	Study period				
	Visit 0—screening visit	Visit 1—baseline visit—Day 2/3 of the first menstrual cycle	Visit 2—Day 3/4 of the first menstrual cycle	Visit 3—Day 2/3 of the third menstrual cycle	Visit 4—oocyte pick up day of next IVF cycle
Enrollment					
Eligibility screen	X	X			
Informed consent		X			
Demographic information		X			
Intervention					
Initiation of Shodhana/cleansing (for a period of 18–21 days) followed by Shamana (internal medicines) for a period of 1 month at Amrita school of Ayurveda			X		
Assessments					
Serum AMH at Amrita fertility centre				X	
Ultrasound scan for AFC at Amrita fertility centre				X	
Collection of follicular fluid at Amrita fertility centre					X
Adverse events monitoring(throughout the intervention period)			X		

AFC, antral follicle count; AMH, anti-mullerian hormone.

### Eligibility

Participants will be eligible for enrolment if they meet the following requirements:

#### Inclusion criteria

- Women aged 25–40 years
- Who are willing to provide written informed consent to participate in the clinical trial
- A confirmed diagnosis of DOR fulfilling at least two of the Bologna criteria, namely:

- an abnormal ovarian reserve test, defined as an AFC of <5–7 follicles and/or AMH level of <0.5–1.1 ng/mL; and
- a history of poor ovarian response, defined as retrieval of ≤3 oocytes in a previous IVF cycle.

#### Exclusion criteria

- Diagnosed of genetic disorders
- Undergoing treatment for malignancy
- History of bilateral/unilateral oophorectomy or severe systemic illness
- Participation in other clinical trials
- Contraindications to the Ayurvedic procedures.

## Objectives

Hypothesis: an Ayurvedic treatment protocol might be able to change the ovarian reserve in women diagnosed with DOR, and the pharmacological mechanisms could be investigated through multi-omics approaches.

Exploratory primary objectives:

1. To evaluate the role of an Ayurveda treatment protocol in improving serum AMH levels and AFC in DOR patients.

Exploratory secondary objectives:

1. To perform transcriptomics of granulosa cells (GC) in human follicular fluid through RNA sequencing approaches.
2. To identify therapeutic targets of ayurvedic formulations in improving AMH levels through mass spectrometry-based quantitative proteomics.
3. To identify the key metabolites involved in improving serum anti-mullerian hormone levels through untargeted metabolomics of human follicular fluid.

## Outcomes

Exploratory primary outcome

1 (a). Change in serum AMH.

Time frame:

- Assessment before treatment (BT)—on day2/3 of the first menstrual cycle during recruitment
- post assessment (AT)—on Day 2/3 of the third menstrual cycle that comes after 2 months of Ayurvedic treatment

1 (b). Improvement in ultrasonography count of ovarian antral follicles, AFC.

Time frame:

- Assessment before treatment (BT)—on day2/3 of the first menstrual cycle during recruitment
- Post assessment—on Day 2/3 of the third menstrual cycle that comes after 2 months of Ayurvedic treatment

Exploratory secondary outcomes.

Multi omics analysis of follicular fluid—(Treatment group—10; after 2 months of Ayurveda treatment and Control group—10).

Time frame: day of oocyte retrieval of the IVF cycle.

2. The genes involved in improving the treatment outcomes can be determined through transcriptomics analysis.
3. Through quantitative proteomics, the altered protein profiles can be estimated.
4. Metabolomics approaches enable in the identification of key metabolites involved in improving treatment outcomes in DOR patients.

## Methodology

### Ayurvedic interventions

Women diagnosed with DOR and satisfying the eligibility criteria will be recruited at Amrita Fertility Centre. After due consent, these participants will be subjected to Ayurvedic treatment protocol at Amrita School of Ayurveda.

The intervention protocol begins with śodhana karma (cleansing therapy) and śamana karma (medicines for restoring normalcy or pacifying action). The medicines and formulations selected in the protocol will be individualized according to the participant’s clinical status but follows a standardized therapeutic framework (Table 2).

**Table 2:** Treatment protocol.

Treatment protocol and interventions—Śodhana karma (cleansing therapy)		
Treatment procedure	Method of administration	Treatment duration
Dīpana, pācana (stimulating digestive fire or appetizing action) with Vaiśvānara cūrṇa (24)	5 g twice daily before food with warm water	Day 1–2
Snehapāna (internal oleation). with appropriate ghee or oil	Ārohaṇakrama (gradually increasing dose) after assessing the agni (digestive, metabolic factors) and koṣṭha (bowel habit)	Day 3–8
Sarvāṅga abhyaṅga and svedana (Oleation and sweating treatment)	Sarvāṅga abhyaṅga (application of oil on whole body) with Dhanvantara taila followed by nāḍīsveda (sweating induced by steam directed from hose)	Day 9–10 for vamana Day 9–day 11 for virecana
Either Vamana (therapeutic emesis) or virecana (therapeutic purgation) (25)	Avipatti choorna 30 g given at 7 a.m. for Virecana	Vamana on Day 10 and Virecana on Day 11
Yogabastiḥ (course of eight combined therapeutic enemas)	Three Nirūhabasti (therapeutic decoction enema) with 750 mL of Mustadiyapanabasti and five anuvasanabasti (therapeutic oil enema) with 150 mL of Mahānārayaṇataila administered on alternate days (26)	Day 13–20
Uttarabasti (administration of therapeutic oil or ghee via vagina)	With 3 mL Mahānārayaṇataila into the uterine cavity on the day of Nirūhabasti	
Śamana karma (medicines for restoring normalcy or pacifying action)		
Medicine	Dose	Time and route of administration
Kalyanaka Ghrita/Phala sarpi (medicated ghee) (27)	10 mL	Morning in empty stomach orally
Mahanarayana taila (medicated oil) (28)	10 mL	Evening in empty stomach orally
Satapushpa choorna/Hinguvachadi choorna (medicines in the form of powder) (29, 30)	5 g	Twice daily before food mixed with ghee in the morning and oil in the evening, orally

Śodhana karma (cleansing therapy): the participant will be admitted for a maximum of 21 days following the cessation of menstrual bleeding in the first menstrual cycle. The trial intervention begins with dīpana (stimulating digestive fire or appetizing action) and pācana (digestive action) by administering Vaiśvānara Cūrṇa for 2 days. This is intended to enhance the digestive capacity of the participant and prepare them for the subsequent procedure, snehapāna (internal oleation). Snehapāna (internal oleation) is done by the oral administration of medicated ghee or oil in gradually increasing doses until signs of adequate oleation or samyak snigdha lakṣaṇas are observed or for up to a maximum duration of 7 days. The selection of medicine, dose and duration of snehapāna (internal oleation) will be decided based on the agniḥ (digestive, metabolic factors), koṣṭhaḥ (bowel habit) and clinical condition of the participant. Adequate oleation ensures that the body is properly lubricated for detoxification. Snehapāna (internal oleation) is followed by svedana (sudation therapy) which will be performed for 2–3 days which further facilitates the śodhana karma (cleansing therapy). These preparatory procedures together constitute the pūrvakarma (preparatory phase) of śodhana karma (cleansing therapy).

Depending on clinical indications, participants will then undergo vamana (therapeutic emesis) or virecana (therapeutic purgation), which constitute the main śodhana (cleansing) procedures.

In the concluding part of the śodhana karma (cleansing therapy), participants will receive basti karma (therapeutic enema), which involve medicated enemas. Two forms will be administered:

- Yogabasti (course of eight combined therapeutic enemas)—a combination of five snehabastiḥ (therapeutic oil enema) and three nirūha basti (therapeutic decoction enema) given alternately over 8 days.
- Uttarabasti (administration of therapeutic oil or ghee via vagina)—will be performed on the same day as the nirūha Basti, aimed at directly nourishing and rejuvenating the reproductive organs and their functions.

Śamana karma (medicines for restoring normalcy or pacifying action): following cleansing, participants will begin the śamana cikitsā, consisting of orally administered Ayurvedic formulations for 1 month. These formulations are specifically intended to improve ovarian function and reproductive health by restoring ārtava upadhātu (the supportive component of reproductive system). The selection of medicines will be based on the condition of the patient. All medicines used during the treatments will be procured from a GMP-certified company. To ensure uniformity, the project research scientist who is a specialist doctor will be adhering strictly to the standard operating procedures of the study centre. Participant adherence to the Ayurvedic treatment protocol will be closely monitored throughout the study period using patient file, medication diary, weekly telephonic reminders and digital communication. After undergoing Ayurveda treatment for approximately 2 months, participants will be sent back to Amrita Fertility Centre for post-assessment and further fertility treatments, during which follicular fluid will be collected for multi-omics analysis.

### Adverse events and safety evaluation

All adverse events (AEs) will be documented in the patient file and Case Record Form (CRF) by a trained project research scientist daily during intervention procedures and over phone or on every visit after that. Any associated diseases, conditions or symptoms that present at screening and will persist throughout the intervention without any change will not be considered as an adverse event and will be documented at the time of screening. Laboratory investigations as a part of safety evaluations will involve routine blood and urine investigations prior to initiation and upon completion of the treatment protocol or as and when required along with periodic clinical monitoring of vital signs and general physical health. Participants will be asked to report any new AE/symptoms at any time during the trial. Any reported or observed AE as per CTCAE v5.0 ≤ Grade 2 will be managed by the supportive care and intervention will be continued if it is not persisting after that. Expected AEs and its management are detailed in Table 3. All reported AE ≥ 3 will be reported to principal investigator and the ethics committee.

**Table 3:** Expected adverse events and management.

Procedure/intervention	Expected adverse events	Management plan
Snehapāna(internal oleation)	General weakness, nausea, diarrhoea, abdominal pain, headache, belching and bloating, intolerance to snehapāna	Administering dried ginger water and Aṣṭacūrṇa as a rescue medicine for enhancing the digestion of sneha, adjusting the subsequent dose of snehapāna
Vamana (therapeutic emesis)	General weakness, diarrhoea, dehydration, hypotension, dizziness, mild abdominal discomfort, and loss of appetite	Hydration, diet plan, which is easily digestible, rest
Virecana (therapeutic purgation)	Mild abdominal cramping, dizziness, hypotension, excessive purgation, nausea and vomiting	Adequate hydration with warm water, buttermilk and rice water as rescue measures
Yoga basti (course of eight combined therapeutic enemas)	Abdominal cramping, rectal irritation, incomplete evacuation	Sthānika abhyanga and swedana (massage with oil over abdomen and pelvis) and oral hydration
Uttara basti (administration of therapeutic oil or ghee via vagina)	Mild pelvic discomfort, abdominal cramps, bloating, mild injury and spotting from cervix, hypotension, pelvic infection	Abhyaṅgaḥ and svedana (application of oil and sudation over lower abdomen), oral hydration, Yaṣṭimadhu taila yoni pichu (vaginal tampoon soaked with Yaṣṭimadhu taila inserted in the vagina) as a rescue measure for cervical injury, yoniprakṣālana (medicated vaginal douche) in case of infection
Oral medications	Mild abdominal discomfort, nausea, diarrhoea, anorexia	Water boiled with dried ginger water, temporary dose modification and diet plan which is easily digestible

The intervention will be discontinued if:

- The participant experiences any Grade ≥3 AE
- Repeated Grade 2 AEs persist despite appropriate management.
- The participant withdraws consent at any stage.
- Occurrence of pregnancy.

Participant safety will be monitored continuously by the principal investigator and the study team. Oversight will be provided by the Institutional Ethics Committee, which retains the authority to recommend continuation, modification, or termination of the study if significant safety concerns arise. The invasive procedures like Uttara basti and Yoga basti will be done only under the governance of project research scientist who holds a post graduate degree and received proper training. All aseptic precautions will be taken prior to the procedure. Participants will be confirmed of immunized to Tetanus before invasive procedures like Uttara basti. Any AEs reported during or after the procedure will be reported carefully and will be informed to the principal investigator and appropriate measure will be taken immediately. To prevent any potential possibilities of harming the mother and foetus, in case of an unintended pregnancy, all the participants will be given proper counselling to refrain from sexual intercourse throughout the study duration. All the medicines used for the clinical trial will be procured from Amrita Life, a GMP-certified company. To guarantee the authenticity, safety and uniformity, all the batch of trial medications will undergo predefined QC analysis of the manufacturer. The batches meeting all the standards, with a certificate of analysis, will only be used for the clinical trial.

### Multiomics analysis methodology

#### Transcriptomics of granulosa cells

Follicular fluid collected will be aspirated and pooled during the retrieval of oocytes. Granulosa cells will be isolated from the follicular fluid by centrifugation at 700 g for 10 min using percoll-mediated density gradient centrifugation. The layer with the cells will be carefully removed using a pipette and transferred to a fresh tube with the cell storage medium (10% FBS with DMSO). Total RNA will be extracted from the cells using TRIzol reagent. RNA purity and concentration will be assessed using Nanodrop2000 spectrophotometer (Thermo Fischer Scientific) with samples showing A260/280 between 2.0 to 1.8 considered acceptable and outsourced to MedGenome, Bangalore, India for library preparation and RNA sequencing. The quality of the raw reads will be checked using FastQC and MultiQC. Adapter trimming and low quality read removal will be performed using Trimmomatic v0.39 and the resulting clean reads will be aligned to the human reference genome with STAR (v2.7.10a). Gene counts will be obtained with feature Counts (v2.0.3) and analysed using DESeq21.34.0 in R software. Differential gene expression profiles will be analyzed comparing the control and treatment samples. False positives will be analyzed through Benjamini–Hochberg correction method. The adjusted *p*-value (FDR) less than 0.05 will be considered statistically significant. Following normalization, the gene enrichment analysis will be performed. The top upregulated and downregulated genes (≥1.5-fold) will be further validated using qRT-PCR using specific primers for the selected genes. The relative quantification of genes will be performed through comparative CT analysis.

#### Proteomics of follicular fluid

The follicular fluid collected will be subjected to percoll-mediated density gradient centrifugation. The samples will be centrifuged at 700 g for 10 min and the supernatant containing the follicular fluid proteins will be transferred to a fresh tube. Further, protein estimation will be performed using BCA assay. Since quantitative proteomics needs to be performed through in-solution trypsin digestion, the high abundant proteins present in the follicular fluid will be enriched using Proteominer enrichment kit from Bio Rad. The enriched protein fractions will be eluted, and further protein estimation was done using BCA assay. These protein samples will be resolved on a 15% SDS-PAGE gel to see the overall protein profile after protein enrichment.

100 μg of protein samples will be used for in-solution protein digestion. To achieve this, reduction will be performed using dithiothreitol (100 mM, incubated at 56 °C for 45 min) and alkylation using iodoacetamide (250 mM, incubate for 30 min at RT in the dark). The resulting solutions will be digested using trypsin (1:20 ratio), followed by incubation at 37 °C overnight. The digestion process will be terminated after 16 h by adding 0.1% FA. The solution containing the digested peptides will be subjected to TMT labelling (10 plex) for protein quantification (31). The TMT labelled peptide samples will be desalted and subjected to mass spectrometry analysis. The peptides will be resolved through a C-18 reversed phase column (Agilent ZORBAX SB-C18, 75 μm × 150 mm, 5 μm) having a 40 nL enrichment column also. The samples will be infused to the mass spectrometer through a capillary/nano HPLC equipped with a chip cube source. The mass spectrometric data will be collected in positive ionization mode. The mobile phase solvents will be; A-water+ 0.1% formic acid; B-acetonitrile: water in 90:10 ratio. The enzymatic digests will be resolved across the column using a linear gradient of 5–45 %B in 30 min; 45–90 %B in 20 min. The other parameters that will be used for the data acquisition are: MS; 250–3,000 m/z, MS/MS; 50–3,000, collision gas-nitrogen, capillary voltage; 2000 V, dry gas; 5 L/min, fragmentor voltage; 150 V, source temp; 350 °C, MS and MS/MS scan speed; 4 & 3 spectra/s, respectively. Number of precursors; 5, preferred charge states; trypsin (2, 3, >3). The data will be acquired using MassHunter (Version 6.0) software from Agilent Technologies. The raw data obtained after MS analysis will be analysed using MassHunter, Mascot and Proteome Scaffold using the Swissprot database. The following parameters will be set during data analysis: MS tolerance; 10 ppm, MS/MS tolerance; 20 ppm, cysteine (carbamidomethylation; fixed modification), TMT labelling will be also selected as fixed modification. Deamidation of asparagine and glutamine will be used as variable modifications, number of missed cleavages; 2, charge states (2^+^, 3^+^, 4^+^) for trypsin, decoy option will be enabled during the analysis. Proteome Scaffold will be used to statistically validate the proteins and peptides identified through Mascot searches as described under the statistical methods section. Protein data will be filtered at 5% FDR and normalized for downstream quantitative analysis. The differentially expressed proteins will be selected after the quantification and selected top proteins (≥1.5-fold) will be further validated through ELISA and Western blotting using the specific primary antibodies using the same follicular fluid samples.

### Metabolomics of follicular fluid

For metabolomics analysis, the metabolites will be extracted using a solvent mixture containing isopropanol: acetonitrile: water (2:1:1). Four volumes of the cold solvent will be added to one volume of sample. The samples will be homogenized by mixing and gentle shaking. The proteins will be precipitated by incubating the mixture at −20 °C for 1 h. After centrifugation (9,000 g/15 min/4 °C), the organic layer will be carefully removed and transferred on to a new tube. The tube will be subjected to flash evaporation using nitrogen and dried completely. A solution containing 5% acetonitrile with 0.1% formic acid will be used for redissolving the dried organic phase and further subjected to LC–MS/MS analysis. The analyses will be performed on a C18 column (Agilent Zorbax; 150 × 2.1mm, 2.7 μM) through a uHPLC system. LC–MS/MS analysis will be performed using a Q-TOF mass spectrometer (Agilent Technologies) equipped with the Dual AJS source. Infusion of reference mass ions (118.0862, 322.0481, 922.0097, 2121.9331) will ensure high mass accuracy over a wider mass range. 100% water with 0.1% formic acid will be used as mobile phase A and 100% acetonitrile with 0.1% formic acid will be used as mobile phase B. A liner gradient of 5–95% B for 45 min and a flow rate of 200 μL/min will be set, and the peaks will be monitored at 254 nm using a diode array detector. MS data will be acquired in both positive and negative ionization mode with a fixed collision energy. The other parameters that will be used for the MS data acquisition includes; nebulizer pressure; 15.0 psi, dry gas flow: 10 L/min, dry gas temperature; 350 °c, scan range; 50–2000 m/z, no of precursors; 5.

**Figure 2:**
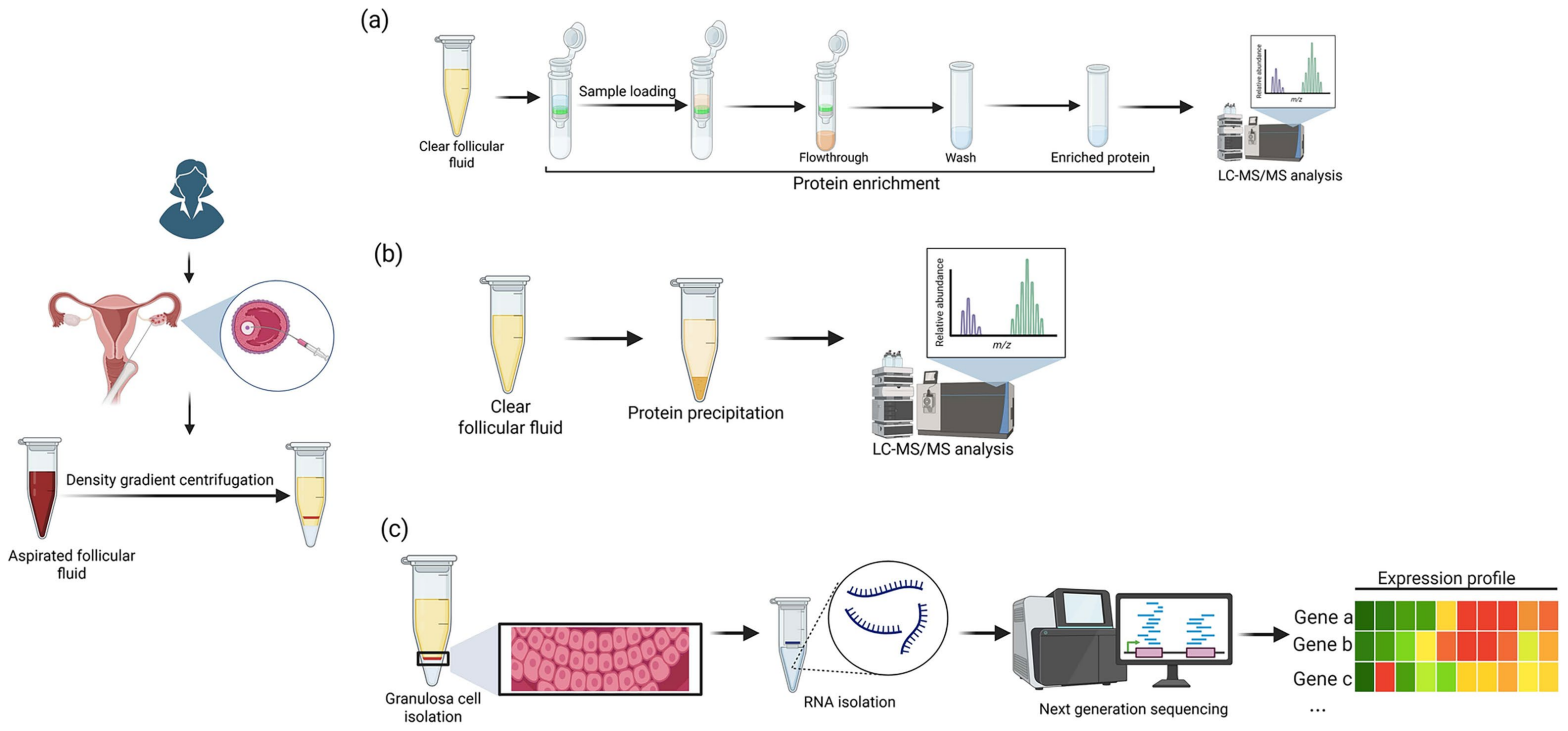
The overall workflow for **(a)** proteomics **(b)** metabolomics and **(c)** transcriptomics analysis. (Created in BioRender. Nair, B. (2025) https://BioRender.com/gux0gyw).

The raw data will be processed using Agilent MassHunter qualitative analysis software (Version 7.0) enabling the molecular feature extraction module. The data obtained will be aligned using their respective retention time and m/z values. The molecular feature extraction workflow enables in the identification of the respective compounds through aligning the respective retention time and m/z values. Subsequently, the elemental composition analysis (of monoisotopic m/z or the average isotopic m/z values) will be performed using the formula finder tool in the MassHunter software. The data will be further filtered to make sure like only validated and high-quality data will be further taken for the analysis. The intensity threshold will be set for minimizing the influence of signal to noise ratio for the accurate identification. The metabolites present will be identified incorporating all the stringent conditions including retention time, monoisotopic masses, ion intensity. Followed by this, the information of all the compounds will be identified and searched using the Agilent MassHunter PCDL (Personal Compound Database and Library). For metabolome analysis, the collected data, after filtering and extraction process will be searched using PubChem (National Center for Biotechnology Information), ChemSpider, Agilent Metabolomics Lab, METLIN (Scripps Center for Mass Spectrometry), KEGG (Kyoto Encyclopedia of Genes and Genomes) and HMDB (Human Metabolome database). The structure and identity will be viewed through the ‘compound find’ tab in the MassHunter qualitative analysis software. Finally, heatmaps, principal component analysis and multivariate statistical analysis will be performed using ‘R’ and Bioconductor package. All the metabolite data information collected will be also normalized using MetaboAnalyst 6.0. Principal component analysis will be used to evaluate the quality, identify outliers, and display global patterns in the metabolomic data of follicular fluid. Through MetaboAnalyst, supervised modelling will be carried out using partial least squares–discriminant analysis (PLS-DA) to find metabolite signatures that differentiate DOR participants from controls. A nested cross-validation will be performed using an outer five-fold loop and inner three-fold loop for estimating unbiased performance and for feature selection, respectively, as indicated in the MetaboAnalyst pipeline. To cross validate the model performance, root mean squared error (RMSE), *R*^2^, and permutation testing will be done. Further, the differentially regulated metabolites (fold change ≥1.5) will be evaluated using Benjamini–Hochberg (5% FDR) correction, and features that are chosen several times will be given higher priority as strong candidates. These metabolites will be finally correlated with the obtained AMH and AFC data. Figure 2 illustrates the overall multi-omics workflow adopted in the study.

### Integration of multiomics data

The transcriptomics, proteomics and metabolomics data will be processed separately. The differentially expressed genes compiled after transcriptomics analysis will be studied in detail through ingenuity pathway analysis to study the upstream regulators, signalling pathways involved. Similarly, KEGG analysis will be used to determine the biological pathways that are enriched with differentially expressed proteins and metabolites. Finally, Multi-Omics Factor Analysis (MOFA) will be performed using the MOFA2 package in the Bioconductor software package (release 3.21). For this, the multiomics data obtained will be trained and analyzed to correlate with the AMH and AFC changes at 1% FDR with BH correction. Significant genes, proteins, and metabolites will be mapped onto biological pathways or networks, and correlations with clinical outcomes (AMH/AFC changes) will be explored to highlight mechanistically relevant associations.

## Data collection

On recruitment, participant consent will be obtained and documented in an Informed consent form (ICF) prepared in both Malayalam and English language. The basic demographic data, detailed medical history, baseline assessment details and all other relevant data will be collected and documented in an analog case report form (CRF). These will be done by the project research scientist at Amrita School of Ayurveda.

Clinical data collection: The baseline and post intervention clinical data for analysis will be collected through a blood test and ultrasound scan by experts at Amrita Fertility Centre and laboratory associated with the centre. To ensure consistency, reduces inter observer variability and improves reliability the same radiologist will perform the sonography and laboratory samples will be analysed at the same lab. To ensure quality assurance and reproducibility, standard operating procedures (SOPs) will be followed at every level of data collection and handling. Laboratory technician evaluating the biological samples and radiologist, will continue to be blind to the treatment schedules and clinical results to further enhance scientific rigour.

Follicular fluid collection: The follicular fluid will be collected from 10 participants who visit Amrita Fertility Centre after Ayurvedic treatment. Follicular fluid from 10 participants who did not avail of Ayurvedic treatment protocols undergoing IVF treatment will also be taken as control. During oocyte retrieval procedure, the follicular fluid will be aspirated from multiple follicles by the principal investigator, a Professor of Reproductive Medicine at Amrita Fertility Centre. The follicular fluid collected, after removing the cell debris through centrifugation (700 g for 10 min), will be immediately transferred to liquid nitrogen contained in the cryocan and transported to Amrita School of Biotechnology, by the project research scientist who is trained on handling and pre-processing the follicular fluid samples for performing multi-omics analysis. These samples will be transported to Amrita School of Biotechnology using a cryocan (with liquid nitrogen). Further, the follicular fluid samples will be divided into multiple aliquots in 5 mL Eppendorf tubes to avoid repeated freeze–thaw cycles and these aliquots will be stored in −80 °C till further analysis.

## Data quality and control

To enable simple and accurate data entry and processing, hard copy of the CRFs will feature numbered and coded items. The principal investigators will review versions of the forms before they are finalized. To guarantee thorough data collection, standard operating procedures along with the templates will be created, and the research scientists working as field personnel will receive the necessary training. This will involve assessing their own performance through quality control, which involves looking for any missing information or improbable answers. Additionally, after data collection, principal investigators will do additional quality control checks to ensure that the collected data are complete, consistent and comprehensive. Before the CRFs are used for data documentation necessary corrections/modifications will be made.

### Standardization of the quality of laboratory and imaging assessments

Serum AMH levels will be measured through electrochemiluminescence immunoassay (ECLIA) on a cobas® e 602 analyzer (Roche diagnostics, Germany). Using Beckman Coulter AMH Gen II ELISA (unmodified version, without predilution), the assay will be standardized prior to use. To ensure accurate calibration and traceability of results, through the relevant CalSet, the predefined master curve will be adapted. The assay calibration will be performed once per reagent lot (using a fresh reagent each time, preferably not more than 24 h since the analyzer has registered using the cobas e pack). This calibration interval will be dependent on the result verification done on each laboratory and for the renewal, certain conditions will be prespecified and recommended before next use, such as, if using the same reagent lot, the assay will be calibrated after 12 weeks, and if same cobas e pack is used in the analyzer, calibration will be done after 28 days. The QC analysis on each lot will be performed using PreciControl AMH. For this, the controls for each of the standard AMH concentrations will be done once every 24 h or once per cobas e pack, following the calibration protocol mentioned above. The validated measuring ranges for the appropriate clinical use will be set as 0.01–23 ng/mL.

The assessment of antral follicles by will be done by transvaginal ultrasonography using a high-resolution ultrasound machine (LOGIQ P5, GE Healthcare, USA) with a transvaginal probe operating at 6–8 MHz. In order to guarantee the consistency, the same ultrasound equipment will be used throughout the study period. To reduce inter-observer variability, a single operator with over 15 years of pelvic ultrasonography experience will perform all scans. As advised by the manufacturer, the ultrasound equipment will receive routine calibration and preventive maintenance.

## Data security

All participant data will be internally documented using unique identifier codes to maintain confidentiality. Access to these identifiers will be restricted to the core research team, including the Principal Investigator, Co-Investigators, and Project Scientists directly involved in the study. Other than the hardcopy the data will be encrypted on a hard drive with a password. None of the details that reveal the identity of the participant will be stored in the database. All data will be accessed using the anonymous codes that are assigned to each participants who are involved in the clinical trial. Additionally, the follicular fluid samples collected from these individuals will be assigned with the same coding and these will be recorded in a paper and stored in the server with passwords. The data collected after the follicular fluid data analysis will be given utmost security, that none of these would be made available in public domain. If the data generated is used for publication process, the identity of the participant will be kept anonymous and none of the personnel data will be shared. However, few data including the participant’s previous IVF history and demographic information will be used in the publication on a case basis. Nevertheless, all the sensitive information regarding the participant and the samples collected will be secured.

## Data storage

After completing the study, all the files related to the study will be stored in a server and in an encrypted hard drive. The analog CRFs will be kept locked in cupboards for storing it for long term. Both the electronic and hard copy of all the data will be stored for a minimum of 10 years after the study completion and frequent checks will be done to ensure that the data is accessible and available. The multiomics data of the collected samples will be stored in a encrypted storage device. The raw proteomics mass spectrometry data will be submitted to ProteomeXchange consortium (32) via the PRIDE (33) partner repository. After submitting the raw data, a dataset identifier number will be provided, and it will be available with a accession number in the PRIDE repository.

## Statistical methods

The AMH and AFC changes within the 30 participants who underwent Ayurveda treatment will be assessed using paired *t*-tests (if the data is normally distributed) or Wilcoxon signed-rank test (if the data is non-normal), as determined by Shapiro–Wilk test. Statistical significance is considered as *p* < 0.05 for all two-sided tests. Results will be reported as mean ± SD or median (IQR), with effect sizes (Cohen’s d) for pre-post changes. As a sensitivity analysis, ANCOVA will be utilized for adjusting post-treatment data for baseline AMH/AFC and variables such as age, BMI, and ovarian response.

Follicular fluid from 10 control participants and 10 Ayurvedic-treated participants chosen from the 30-participants treatment arm and matched according to age and baseline AMH/AFC will be obtained for the secondary, exploratory multi-omics analysis. All the laboratory experiments will be performed in triplicates (of each biological samples) and statistical analysis will be done using GraphPad Prism software. The statistical analysis of collected proteome data will be performed using Proteome Scaffold software (version 4). Peptide identifications will be accepted if they can be established at greater than 95.0% probability. The Scaffold Local FDR algorithm and peptide prophet algorithm with scaffold delta-mass correction will assign peptide probabilities. Protein identifications will be accepted if they can be established at greater than 99.0% probability and contain at least two peptides representing the protein family. The protein prophet algorithm will assign protein probabilities (34, 35). For metabolomics analysis, after data generation, the heatmaps, principal component analysis, and multivariate statistical analysis will be performed using R (Version 4.5.1) and MetaboAnalyst 6.0. To evaluate the differences between the treatment and control groups in transcriptomics, proteomics, and metabolomics, *t*-tests (for normally distributed data) or Mann–Whitney U tests (for non-normal data) will be used for each gene, protein, or metabolite. To account for multiple comparisons, the Benjamini–Hochberg FDR correction will be used; an FDR of less than 0.05 will be considered significant. As described under each section, specific assays will be used to further validate the most important genes and proteins. No formal sample size calculation was performed for the exploratory multi-omics analysis, as it is hypothesis-generating, and the 10 treatment samples were selected based on matching and feasibility.

## Discussion

Diminished ovarian reserve characterized by reduction in oocyte quality and number often results in poor reproductive outcomes, posing a great challenge in successful assisted reproductive techniques. It was observed that DOR is the most common reason for selecting ART in fertility treatments (4). Women with DOR tend to exhibit poor ovarian response to gonadotropins, requiring higher doses of medication and prolonged stimulation protocols, yet still yielding suboptimal results. Even though various stimulation protocols (36) and other options are being tried, patients become poor responders due to low quality or quantity of oocytes. Currently, there is no established standard of care that can effectively restore the ovarian reserve, hence underscoring the need for novel and integrative treatment approaches. In recent years, traditional medicine systems like Ayurveda, Traditional Chinese medicine have attracted scientific interest for their potential in enhancing and improving various aspects thereby alleviating the disease conditions including PCOS and DOR. The importance of investigating the role and impact of traditional medicines has been highlighted through a study that used an Ayurveda treatment protocol in improving AMH values in DOR conditions (13). Another study has highlighted the importance of traditional Chinese medicine that includes herbal treatment along with acupuncture in improving the AFC and FSH levels in women with DOR (37). Similarly, the cell signalling pathways modulated in granulosa cells using Er Zhi Tian Gui Formula suggested its potential (38). The impact of few formulations including Gengnianchun and Bushen Jianpi Tiaoxue Decoction on mTOR and estrogen signaling pathways in reducing apoptosis and oxidative stress in granulosa cells has been demonstrated (39, 40). A recent meta-analysis has also shown the impact of certain East Asian interventions including herbal and acupuncture therapies in improving the live birth and clinical pregnancy rates with minimal adverse events (41). Following these approaches, this study protocol aims to investigate the feasibility and potential of an Ayurvedic treatment in improving the ovarian reserve markers in DOR patients. Through integrative approaches this study might be able to predict the advantages of traditional Ayurvedic treatment approaches in improving the ovarian reserve markers and the overall ovarian microenvironment.

### Limitations

Since this is a single-arm clinical trial, the primary objective is to explore the potential role of Ayurvedic treatment in women with DOR by assessing changes in AMH and AFC. Accordingly, the findings should be regarded as preliminary and hypothesis-generating. Also the results of this study cannot be considered conclusive or generalized without validation through larger, controlled studies. A further limitation is that the evaluation is restricted to surrogate biomarkers, namely AMH and AFC. While these provide useful information about ovarian reserve, the impact of the intervention on clinically meaningful outcomes such as oocyte quality, fertilization rate, and clinical pregnancy will not be assessed. Nevertheless, given the exploratory nature of this trial, the focus will be on generating preliminary evidence and mechanistic insights into the effects of Ayurveda-based interventions in DOR. The knowledge gained is expected to inform and guide the design of future controlled studies that will incorporate broader reproductive and clinical endpoints.

### Confidentiality

All the study related patient information and the results will be stored securely at the Amrita School of Ayurveda and Amrita School of Biotechnology. The files pertaining to the study will be securely locked in cupboards with limited access. The trial participants will be assigned a code, and this will be used for maintaining the confidentiality of the patient records and sample analysis results. None of the personnel information or identifying information will be stored along with the participant’s records. The data will be also secured in local databases with password protection.

### Dissemination plan

The clinical trial is registered in the clinical trial registry of India (crri.nic.in). The time of starting and finishing the recruitment details will be updated in the registry. After completing the data collection, the results and reports including the workflow, the demographic information of the trial participants, primary outcomes and the statistical analysis will be updated. This will be made available to the respective stakeholders including interested participants and IVF centres. The outcome of the study will be published in peer-reviewed international journals and the work will be presented in conferences and seminars. As recommended by International Committee of Medical Journal Editors, will follow all the guidelines and criteria while preparing abstracts and publications. The findings may serve as a basis for future clinical trials on Ayurvedic interventions in ART.
